# CCIDB: a manually curated cell–cell interaction database with cell context information

**DOI:** 10.1093/database/baad057

**Published:** 2023-08-11

**Authors:** Jin Young Noh, Hae In Lee, Ji-Hye Choi, Seong-Ho Cho, Yoon Hui Yi, Ji Hyun Lim, Eun Been Myung, Ye Ju Shin, Hyo Jin Shin, Hyun Goo Woo

**Affiliations:** Department of Physiology, Ajou University School of Medicine, Suwon 16499, Republic of Korea; Department of Biomedical Science, Ajou University Graduate School, Suwon 16499, Republic of Korea; Ajou University Data Center for Biomedicine & Pharmacotherapeutics (AUDC-BMPT), Ajou University School of Medicine, Suwon 16499, Republic of Korea; Department of Physiology, Ajou University School of Medicine, Suwon 16499, Republic of Korea; Department of Biomedical Science, Ajou University Graduate School, Suwon 16499, Republic of Korea; Ajou University Data Center for Biomedicine & Pharmacotherapeutics (AUDC-BMPT), Ajou University School of Medicine, Suwon 16499, Republic of Korea; Department of Physiology, Ajou University School of Medicine, Suwon 16499, Republic of Korea; Department of Biomedical Science, Ajou University Graduate School, Suwon 16499, Republic of Korea; Department of Physiology, Ajou University School of Medicine, Suwon 16499, Republic of Korea; Department of Biomedical Science, Ajou University Graduate School, Suwon 16499, Republic of Korea; Ajou University Data Center for Biomedicine & Pharmacotherapeutics (AUDC-BMPT), Ajou University School of Medicine, Suwon 16499, Republic of Korea; Ajou University Data Center for Biomedicine & Pharmacotherapeutics (AUDC-BMPT), Ajou University School of Medicine, Suwon 16499, Republic of Korea; Ajou University Data Center for Biomedicine & Pharmacotherapeutics (AUDC-BMPT), Ajou University School of Medicine, Suwon 16499, Republic of Korea; Ajou University Data Center for Biomedicine & Pharmacotherapeutics (AUDC-BMPT), Ajou University School of Medicine, Suwon 16499, Republic of Korea; Ajou University Data Center for Biomedicine & Pharmacotherapeutics (AUDC-BMPT), Ajou University School of Medicine, Suwon 16499, Republic of Korea; Department of Physiology, Ajou University School of Medicine, Suwon 16499, Republic of Korea; Department of Biomedical Science, Ajou University Graduate School, Suwon 16499, Republic of Korea; Ajou University Data Center for Biomedicine & Pharmacotherapeutics (AUDC-BMPT), Ajou University School of Medicine, Suwon 16499, Republic of Korea

## Abstract

Cell–cell interaction (CCI) is a crucial event in the development and function of multicellular organisms. The development of CCI databases is beneficial for researchers who want to analyze single-cell sequencing data or study CCI through molecular experiments. CCIs are known to act differently according to cellular and biological contexts such as cell types, gene mutations or disease status; however, previous CCI databases do not completely provide this contextual information pertaining to CCIs. We constructed a cell-cell interaction database (CCIDB) containing the biological and clinical contexts involved in each interaction. To build a database of cellular and tissue contexts, we collected 38 types of context features, which were categorized into seven categories, including ‘interaction’, ‘cell type’, ‘cofactor’, ‘effector’, ‘phenotype’, ‘pathology’ and ‘reference’. CCIs were manually retrieved from 272 studies published recently (less than 6 years ago). In the current version of CCIDB, 520 CCIs and their 38 context features have been manually collected and curated by biodata engineers. We suggest that CCIDB is a manually curated CCI resource that is highly useful, especially for analyzing context-dependent alterations in CCIs.

**Database URL** https://ccidb.sysmed.kr/

## Introduction

Biosystems are composed of numerous cells, and the interactions between these cells lead to an exchange of important biological signals that play an important role in maintaining biological functions. To date, various types of cell–cell interactions (CCIs), including ligand–receptor interaction, extracellular matrix (ECM)–receptor and receptor–receptor interactions, have been identified. Recent advances in single-cell RNA sequencing (scRNA-seq) technology have enabled us to distinguish between cell types and estimate their transcription. Single-cell-level profiling can reveal CCIs at the single-cell level (1). Previously, several databases for CCI, such as BaderLabDB (2), LRdb (3), CellPhoneDB (4) and CellChatDB (5), have been developed using either the interactions reported in the literature or computationally predicted interactions. For example, BaderLabDB (2) contains 115 900 ligand–receptor (LR) interactions found in humans. LRdb (3) contains 3251 human LR interactions. CellPhoneDB (4) contains 930 human LR interactions. CellChatDB (5) contains 3960 interactions documented in humans and mice. These databases contain useful information about CCIs such as the source gene, target gene, interaction name, reference features, including ‘db resource’, ‘db source’ and ‘PubMed ID (PMID)’ and information about the cell type, including ‘species’. These CCI databases are being actively used as a resource by researchers to understand and study the mechanisms underlying cellular phenomena and physiological functions regulated by interactions between cells. Moreover, dysregulated CCIs can lead to the development and progression of diseases; therefore, understanding CCIs can help researchers in gaining deep insights into the pathobiology of diseases.

CCIs are differentially regulated based on cell types and cellular conditions such as gene mutations, disease status and other genetic perturbations. These cellular contexts affect CCIs, regulating their downstream cellular effects such as those on cell growth, death, differentiation and disease development and progression (6). Nevertheless, previous CCI databases have not incorporated well-established CCIs that act differentially depending on the cellular context. Indeed, malignant cells interact with fibroblasts, immune cells and other tissue cells; however, their interactions can be differentially regulated depending on the cellular context, such as mutations, gene expression levels or grades of disease progression. For example, tumor cells with *TP53* mutated that interact with fibroblasts contribute to tumor progression by mediating angiogenesis (7). Moreover, fibroblasts in cancer tissues, which show different cellular characteristics, are called cancer-associated fibroblasts (CAF). CAFs secrete IL-6, interact with IL-6R and facilitate cancer cell progression (8). These findings reveal that even with the same cell type, CCI may differ depending on the disease status and cellular context. Therefore, we suggest that the cellular context should be considered for CCI analysis.

In this study, we developed a cell-cell interaction database (CCIDB), containing the cellular contexts that are important for cell functions such as cell type, pathology and phenotypes. We manually retrieved and curated the information pertaining to context-dependent CCIs from recently published literature and compared it with previous CCI databases. We demonstrated that our database is beneficial for biological interpretation as well as for understanding context-dependent underlying mechanisms.

## Results

### Construction of CCIDB with context information

We gathered 272 literature studies on CCIs from biomedical journals with an impact factor higher than 5 and published within the past 6 years; we then manually retrieved 520 CCIs and related information. The interactions were reviewed and manually curated by biodata engineers with bachelor’s degrees in biology-related subjects.

To construct a CCI database containing cellular context-related information, we collected 38 features for the cell context from the literature. We categorized the context features into seven categories, including ‘interaction’, ‘cell type’, ‘cofactor’, ‘effector’, ‘phenotype’, ‘pathology’ and ‘reference’. The complete list of features is presented in Table 1. The ‘interaction’ category included the interaction features such as ‘interaction type’, ‘interaction name’ and ‘signaling type’. The ‘interaction’ feature had the names of genes involved in the CCI, i.e. ‘source gene’ and ‘target gene’, which we used as the official gene symbols for humans (HUGO) (9) and mice (MGI) (10). In cases where an official gene symbol was not available, we adopted the terminology used in the corresponding literature to refer (i.e. Acetylcholine, Lipid and Polyunsaturated fatty acid). The ‘interaction type’ included ‘Ligand–receptor’, ‘ECM–receptor’ and ‘Receptor–receptor’. The ‘interaction name’ described the paired source-target genes, and the ‘signaling type’ described paracrine, autocrine and juxtracrine signaling. The ‘cell type’ category included the features such as ‘source cell’, ‘species’ and ‘target cell’. The nomenclatures from the human protein atlas (11) were used as cell type names. The ‘cofactor’ category included ‘source gene cofactor’ describing the agonist or antagonist cofactor type. The ‘source gene cofactor function’ and ‘target gene cofactor function’ features described the cofactor functions for the source gene and target gene, respectively. The ‘effector’ category included features, such as ‘effector’, ‘pathway name’ and ‘effector’s function’, describing the downstream effector genes or pathways and their functions. The ‘phenotype’ category contained ‘mode of action’ and ‘phenotype’ features. The ‘mode of action’ described the functions of the target cells, including activation or inhibition. The ‘phenotype’ feature described several representative cellular phenotypes, such as metastasis, proliferation, angiogenesis, invasion and progression. The ‘pathology’ category described the pathological information of the source and target cells, including the features of ‘tissue’, ‘cell pathology’, ‘cell perturbation’, ‘cell stage’ and ‘patient’s pathology’. The cancer-associated cells, including CAFs, tumor-associated macrophages, tumor endothelial cells and tumor-associated neutrophils, were separately described in the ‘cancer-associated’ feature. The ‘cell perturbation’ feature described experimentally perturbed conditions, including knockdown or overexpression, treatment with reagents and experimental conditions (e.g. hypoxia). The ‘cell stage’ feature described the cell stage such as T cell transition to express CD4 or CD8, or transition of macrophage into M1 or M2. The ‘reference’ category described the information about the reference literature, including ‘DB resource’, ‘DB source’, ‘PMID’, ‘journal title’, ‘journal name’, ‘first author’ and ‘publication year’.

**Table 1.** Comparison of context features included in CCIDB and other CCI databases.

Category	Context feature	CCIDB	CellChatDB	LRDB	CellPhoneDB	BaderlabDB
Interaction	Source gene	O	O	O	O	O
	Source gene alias	O		O		
	Target gene	O	O	O	O	O
	Target gene alias	O		O		
	Interaction name	O	O	O	O	O
	Interaction type	ECM–receptor Ligand–receptor Receptor–receptor	Cell–Cell contact ECM–Receptor Secreted signaling	Ligand–receptor	Ligand–receptor	
	Signaling type	Autocrine Paracrine Juxtacrine				
Cell type	Source cell	O				
	Literature source cell	O				
	Target cell	O				
	Literature target cell	O				
	Species	Human Mouse Bovine Chicken	Human Mouse	Human	Human	Human
Cofactor	Source gene cofactor	O	O			
	Source gene cofactor function	O	O			
	Target gene cofactor	O	O			
	Target gene cofactor function	O	O			
Effector	Effector	O				
	Effector’s function	O				
	Pathway name	O	O			
Phenotype	Phenotype	O				
	Mode of action	O				
Pathology	Source tissue	O				
	Target tissue	O				
	Source cell pathology	O				
	Target cell pathology	O				
	Source cell perturbation	O				
	Target cell perturbation	O				
	Source cell stage	O				
	Target cell stage	O				
	Patient’s pathology	O				
	Literature patient’s pathology	O				
Reference	DB resource	CCIDB	CellChatDB	LRDB	CellPhoneDB	BaderlabDB
	DB source	PubMed	PubMed PMC KEGG	PubMed	PubMed PMC	PubMed
	PMID	O	O		O	O
	Journal title	O				
	Journal name	O				
	First author	O				
	Publication year	O				
Total		38	13	10	8	7

**Figure 1.**
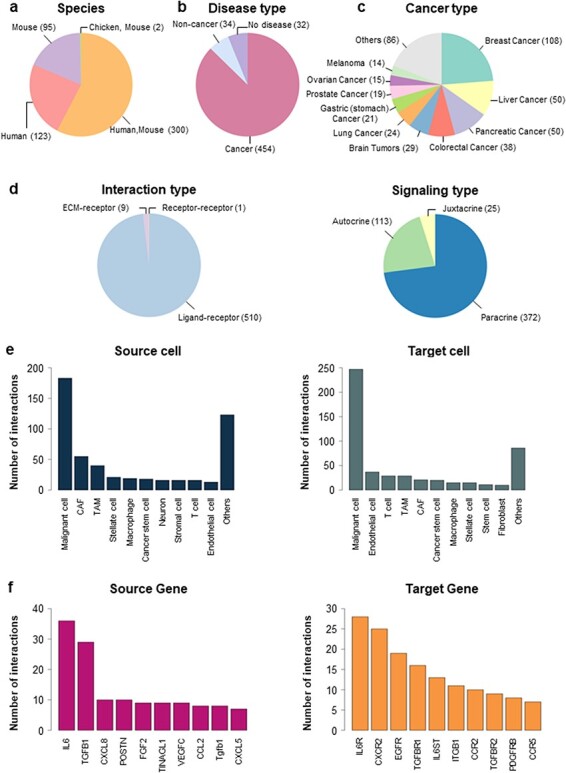
Distribution of CCIDB. (a–d) Pie plots showing the distribution of the features in CCIDB, including species (a), disease type (b), cancer type (c), interaction type (d, *left*) and signaling type of the LR interactions (d, *right*). (e–f) Bar plots showing the top-ranked 10 frequent source cell types (e, *left*), target cell types (e, *right*), source genes (f, *left*) and target genes (f, *right*).

In CCIDB, we manually collected 520 CCIs and their 38 cell context features from 272 studies. Interactions were obtained from different species including humans (*n* = 123), mice (*n* = 95) and combined (*n* = 300) (Figure 1a). Although we tried to select literature in an unbiased manner, many studies included research data on cancers (87.31%, *n* = 454), including breast cancer (*n* = 108), liver cancer (*n* = 50), pancreatic cancer (*n* = 50) and others (*n* = 246) (Figure 1b, c). The interaction types included 510 Ligand–receptor, 1 Receptor–receptor and 9 ECM–receptor interactions (Figure 1d, *left*). Signaling types of LR interactions included 372 paracrine, 113 autocrine and 25 juxtacrine (Figure 1d, *right*).

When we examined the network structure of the cell-to-cell interactions, malignant cells had the highest number of interactions between the source cell (*n* = 183, Figure 1e, *left*) and target cell (*n* = 247, Figure 1e, *right*). In cell-to-cell interaction, malignant cells exhibited the highest frequency of autocrine signaling (*n* = 75, 21.87%), which is a type of signaling where cells produce and respond to their own signaling molecules. Interactions with CAFs (*n* = 61, 17.78%) and TAMs (*n* = 49, 14.29%) were also observed frequently in CCIDB (Supplementary Figure S1). In gene-to-gene interactions, CCIDB included 201 source genes and 199 target genes. Indeed, *IL6* (Interleukin-6) showed the highest number of interactions among the source genes (*n* = 36, Figure 1f, *left*), whereas *IL6R* (Interleukin-6 receptor) showed the highest number of interactions among the target genes (*n* = 28, Figure 1f, *right*). These results imply the functional significance of *IL6* and *IL6R* in the overall CCIs.

### Comparison of CCIDB with previous CCI databases

We compared the context features of CCIDB with those of previous databases, including CellChatDB (5), LRDB (3), CellPhoneDB (4) and BaderLabDB (2) (see Table 1). These databases had the following features in common: ‘source gene’, ‘target gene’, ‘interaction name’, ‘species’, ‘DB resource’, ‘DB source’ and ‘PMID’. In addition to these, our CCIDB had the context features such as ‘cell type’, ‘cofactor’, ‘effector’, ‘phenotype’ and ‘pathology’, which were considered as contexts that significantly affect CCIs, which were not included in the previous CCI databases. In addition, CCIDB contains the results from latest research carried out using the latest single-cell technology within the past 6 years from 2016 to 2021 (Table 2); therefore, we suggest that the CCIDB has the advantage of being up-to-date over the other databases. Indeed, 213 of 520 CCIs (38.65%) are present in our database, but not in previous databases (Supplementary Table S1). For instance, recent studies have shown that CD24 promotes immune evasion by interacting with Siglec-10 in tumors (12), and FGL1-LAG-3 interaction mediates T cell suppression in various cancers (13). These crucial interactions were not previously considered in CCI analysis but are included in CCIDB.

**Table 2.** Frequency of publication years of literatures in CCIDB and other CCI databases.

		Publication year	
Database	Number of literatures	∼2015	2016 ∼ 2021
CCIDB	272	0%	100%
CellChatDB	176	26.1%	73.9%
LRDB	2508	99.6%	0.4%
CellPhoneDB	103	91.3%	8.7%
BaderLabDB	105 751	93.4%	6.6%

### Intracellular communication network analysis using CCIDB in breast cancer

**Figure 2.**
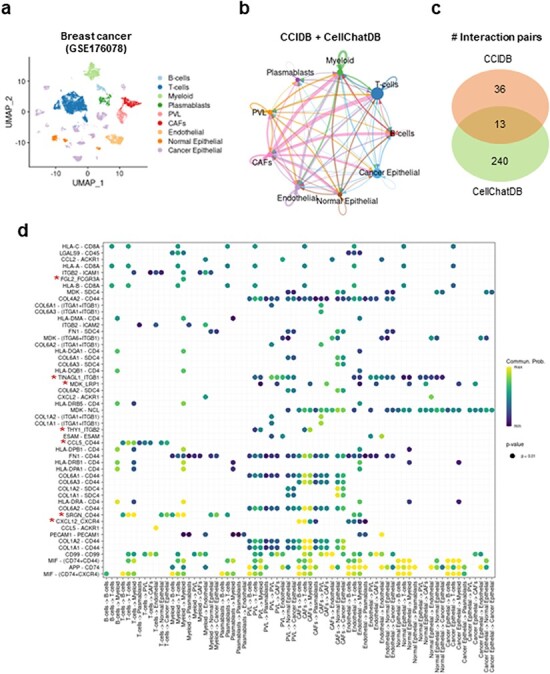
Intracellular communication network analysis using CCIDB in breast cancer. (a) Cell types of the 100,064 cells of breast cancer patients are indicated in a UMAP plot. (b) The inferred intracellular communication network across cell types. Circle size is proportional to the number of cells in each cell group and edge width represents the communication probability. (c) A Venn diagram shows the distribution of interaction pairs identified from CCIDB and CellChatDB. (d) A circle plot shows the interactions of top ranked source-target gene pairs across cell groups. The circle color and size represent the calculated communication probability and *P*-values, respectively. The red asterisks indicate the pairs found in CCIDB.

Next, to assess the utility of our CCIDB, we performed CCI analysis using scRNA-seq data of breast cancer patients (14). After integrating CCIDB and CellChatDB into a reference database, we identified 1610 cell-to-cell interactions and 289 unique significant interaction pairs across 9 major cell types (Figure 2a–b and **Methods**). Indeed, 49 interaction pairs (17.44%, 36 of 281) were detected in CCIDB, while 253 interaction pairs (13.04%, 253 of 1939) were in CellChatDB with 13 overlapped pairs (Figure 2c). When we focused on the top-ranked interaction pairs, we found seven source-target gene pairs from CCIDB (i.e. FGL2_FCGR3A, TINAGL1_ITGB1, MDK_LRP1, THY1_ITGB2, CCL5-CD44, SRGN_CD44 and CXCL12_CXCR4) that showed intracellular communication across various cell types, including T cells, myeloid cells, endothelial cells and cancer epithelial cells (Figure 2d). These results suggest that CCIDB contains CCIs that are relevant in intracellular contexts and were not previously included in other databases.

### Network analysis using CCIDB reveals context-dependent CCI regulators

**Figure 3.**
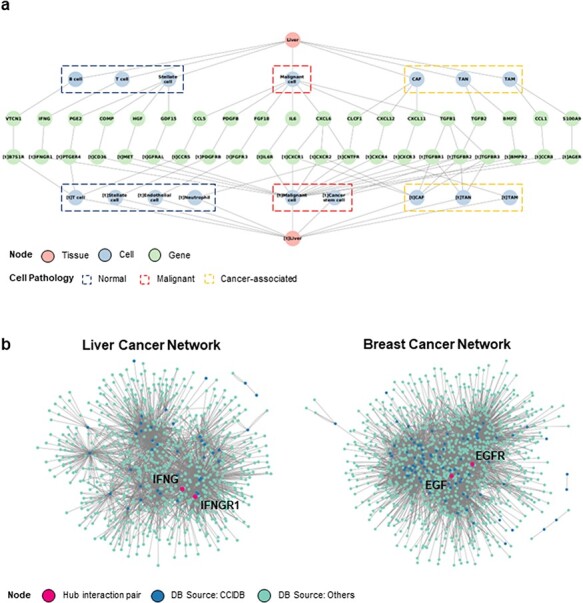
Construction of CCI network. (a) The liver cancer network (LCN) was constructed with six context features, namely ‘source tissue’, ‘source cell type’, ‘source gene’, ‘target gene’, ‘target cell type’ and ‘target tissue’ using Cytoscape. (b) The LCN (*left*) and BCN (*right*) constructed by integrating CCIDB and previous four CCI databases are shown. The source databases of CCIDB (*blue*) and others (*cyan*) are indicated in different colors, and hub pair genes are indicated (*red*).

Next, we performed a context-based network analysis to assess whether context-dependent CCIDB information has a substantial advantage in analyzing the biological significance. We constructed a context-dependent network for liver cancer by restricting the context features (i.e. source/target tissues = ‘liver’, patient’s pathology = ‘liver cancer’) (Figure 3a) that provides the informational CCIs according to the cell type or pathology of source and target cells.

In addition, we merged the CCIDB network with previous DBs (2–5) to improve the limit of the number of interactions. When comparing the CCI networks of liver cancer network (LCN) with the context ‘liver cancer’ and breast cancer network (BCN) with the context ‘breast cancer’, we observed the differential network configurations depending on the context. From the network, we determined a ligand–receptor pair comprising the hub genes (number of interactions >10). In the LCN, we found that *IFNG-IFNGR1* pair had the largest sum of interactions (*IFNG* interactions = 173, *IFNGR1* interactions = 97, total number of interactions = 270). Hence, we suggest that the *IFNG-IFNGR1* interaction plays a regulatory role in the LCN (15) (Figure 2a, *left*). In BCN*, EGF-EGFR* pair was identified as the key regulator (*EGF* interactions = 152, *EGFR* interactions = 358, total number of interactions = 510), which has been known to be associated with poor prognosis in breast cancer (16). (Figure 2a, *right*). Taken together, we suggest that our CCIDB is useful for analyzing context-dependent interactions, which may facilitate the identification of new underlying mechanisms of CCI in disease development and progression.

## Discussion

In this study, we constructed a CCIDB, which includes 38 cellular context features. CCIDB has the most up-to-date information manually retrieved from the literature over the past 6 years (2016–2021), which would be very useful even though the number of interactions is still limited.

The context features included in CCIDB provide detailed cellular information, such as the source and target cell types (e.g. ‘malignant cell’, ‘CAF’ and ‘TAM’) for each interaction, effector genes, their functions (e.g. ‘activation’, ‘inhibition’) and phenotypes (e.g. ‘migration’, ‘invasion’ and ‘angiogenesis’) resulting from the CCI. Additionally, the detailed pathology (e.g. ‘malignant’, ‘cancer-associated’, ‘normal’) and perturbation (e.g. ‘hypoxia’) of the source and target tissues in which the interaction occurs are captured. By utilizing these features, we can construct context feature-based networks to decipher cell–cell communications within specific disease types or among various disease types (see Figure 3).

Previous CCI databases such as LRDB and BaderlabDB were constructed based on literature data mining and did not include information on context features for their interactions. Even the manually curated CellChatDB and CellPhoneDB also did not provide detailed information on cellular phenomena in the interactions. Whereas CCIDB contains data manually curated by biodata engineers with a bachelor’s degree or higher, ensuring more accurate information than predicted data extracted through literature data mining. In fact, we tried to improve the accuracy of the data through a curation process that independently verified the CCI information thrice. Thus, CCIDB provides informative insights into the detailed pathways and effects of intercellular interactions, as well as cellular traits and changes in cellular states. However, to develop a large-scale database in the future, it may also be necessary to obtain additional data using an automated literature mining tool trained with these data.

In conclusion, we suggest that utilizing the contextual information of the CCIDB can help in meaningful data interpretation.

## Methods

### scRNA-seq data analysis

To perform our CCI analysis, we obtained a scRNA-seq dataset of breast cancer patients from the Gene Expression Omnibus (GSE176078). Cells with mitochondria genes expression >20% were excluded. The total number of transcripts in each cell was scaled to 10 000, followed by log transformation. Then, we used Seurat (v4) to detect highly variable genes, perform PCA, graph-based clustering and UMAP. For the analysis, we merged CCIDB and CellChatDB to create an integrated reference database of CCIs. Using CellChat (v1.6.1) (5), we inferred the intercellular communication networks and identified significant interaction pairs based on a cutoff of *P* < 0.01.

### Network analysis

To construct a context-dependent network for liver cancer, we used the context features of tissues, cell types and genes (i.e. ‘source tissue’, ‘source cell’, ‘source gene’, ‘target tissue’, ‘target cell’, ‘target gene’). After filtering the CCIs for liver cancer (source and target tissue = ‘liver’, patient’s pathology = ‘liver cancer’), we constructed a multilayer network according to the context features of source and target genes using by Cytoscape (version 3.9.1). We further expanded the CCIDB networks by merging previous DBs (2–5) and determined a ligand–receptor pair comprising the hub genes (number of interactions >10) for liver cancer and breast cancer, respectively.

## Supplementary Material

baad057_Supp
