# Supplementary material for: CCIDB: a manually curated cell–cell interaction database with cell context information
Source: Database (Oxford). 2023 Aug 11;2023:baad057. doi: 10.1093/database/baad057 (PMC10419333; doi:10.1093/database/baad057)
Supplement: baad057_Supp [file baad057_supp.zip › suppl_data/Supplementary Figure.docx]

## Supplementary Figure.


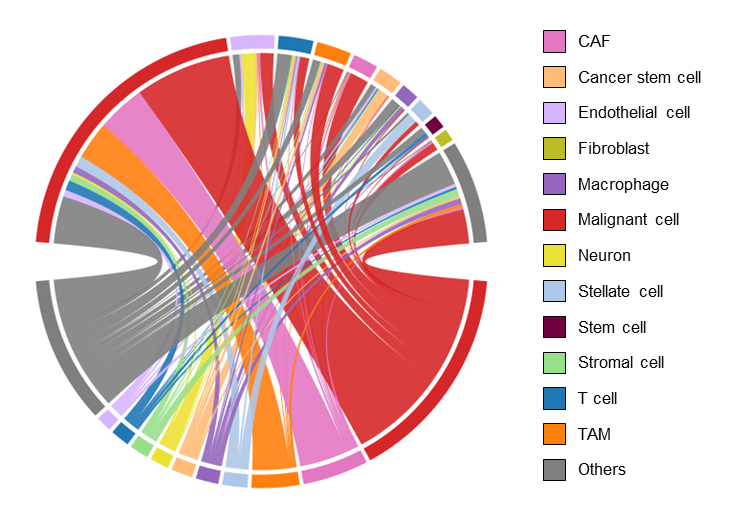


**Supplementary Fig. 1.** **Overview of cell-to-cell interactions in CCIDB.**

A Circos plot showing the interactions between source (*upper circle*) and target (*lower* *circle*) cells for all interactions in the CCIDB.
